# miR-106b-5p targets tumor suppressor gene SETD2 to inactive its function in clear cell renal cell carcinoma

**DOI:** 10.18632/oncotarget.2926

**Published:** 2015-02-24

**Authors:** Wei Xiang, Jun He, Chao Huang, Lejun Chen, Dan Tao, Xinchao Wu, Miao Wang, Gang Luo, Xingyuan Xiao, Fuqing Zeng, Guosong Jiang

**Affiliations:** ^1^ Department of Urology, Union Hospital, Tongji Medical College, Huazhong University of Science and Technology, Hubei Province, Wuhan 430022, China; ^2^ Department of Oncology, The Fifth Hospital of Wuhan, Hubei Province, Wuhan 430050, China

**Keywords:** clear cell renal cell carcinoma, SETD2, miR-106b-5p, p53, proliferation

## Abstract

Inactivation of human SET domain containing protein 2 (SETD2) is a common event in clear cell renal cell carcinoma (ccRCC). However, the mechanism underlying loss of SETD2 function, particularly the post-transcriptional regulatory mechanism, still remains unclear. In the present study, we found that SETD2 was downregulated and inversely correlated with high expression of miR-106b-5p in ccRCC tissues and cell lines. Over-expression of miR-106b-5p resulted in the decreased mRNA and protein levels of SETD2 in ccRCC cells. In an SETD2 3′-UTR luciferase reporter system, miR-106b-5p downregulated the luciferase activity, and the effects were abolished by mutating the predicted miR-106b-5p binding site. Moreover, attenuation of miR-106b-5p induced cell cycle arrest at G0/G1 phase, suppressed cell proliferation, enhanced processing of caspase-3, and promoted cell apoptosis in ccRCC cells, whereas these effects were reversed upon knockdown of SETD2. In addition, transfection of miR-106b-5p antagomir resulted in the increased binding of H3K36me3 to the promoter of p53 and enhanced its activity, as well as upregulated the mRNA and protein levels of p53, and the effects were also abolished by cotransfection with si-SETD2. Collectively, our findings extend the knowledge about the regulation of SETD2 at the posttranscriptional level by miRNA and regulatory mechanism downstream of SETD2 in ccRCC.

## INTRODUCTION

Sporadic clear cell renal cell carcinoma (ccRCC), the most common type of adult kidney cancer [1], is universally characterized by chromosome 3p deletions or loss of heterozygosity (LOH) on chromosome 3p [2]. Three regions were usually involved: (1) 3p25-p26, containing the VHL gene [3, 4]; (2) 3p12-p14.2, harboring the FHIT gene [5–7]; and (3) 3p21-p22, including the PBRM1, PTEN, and SET domain containing protein 2 (SETD2, also known as HYPB) genes [8–10]. Inactivation of these tumor suppressor genes has been demonstrated in previous studies. Undoubtedly, the most frequent event in ccRCC is inactivation of the VHL gene as a result of allelic deletion, somatic mutation and/or promoter hypermethylation, which is well known in familial VHL tumor syndrome and nearly accounts for more than half of the entire cases, including sporadic renal cell carcinoma patients [4, 11–13]. However, increasing evidence indicates that inactivation of VHL alone is not sufficient to initiate ccRCC or maintain the developmental processes of ccRCC [14–16]. Importantly, loss of other tumor suppressor genes has shed additional light on this research topic [3, 14, 17–20], and the most recent studies suggest that dysfunction of tumor suppressor genes on 3p21, such as PBRM1, PTEN, BAP1, and SETD2, might play a vital role in both VHL-dependent or -independent cases [15, 18, 21, 22]. These four tumor suppressor genes may act alone or cooperate with VHL inactivation to initiate tumor formation and/or promote progression.

It is well known that SETD2, a 230-kD protein, plays a key role in Huntington's disease [23, 24]. However, novel information of its other functions has been recently revealed. As an important histone methyltransferase, SETD2 contains a conserved SET domain, which may catalyze histone H3K36 and convert it into trimethylated H3K36 (H3K36me3) [25, 26]. In addition, H3K36me3 presents its association with hyperphosphorylated RNA polymerase II, which is involved in the transcriptional elongation or splicing of specific target genes, and may further change the chromatin structure, which may affect transcriptional activation, DNA repair, and cell cycle regulation, among other processes [25, 27]. Previous reports have indicated that the SETD2 gene may play a tumor suppressor role in some types of cancer; for example, it was reported that a lower transcript level of SETD2 was found in breast cancer tissues and that the development of the tumor was correlated with reduced SETD2 mRNA levels [28, 29]. Remarkably, inactivation of SETD2 has been found in cell lines of ccRCC and clinical samples in which SETD2 has been demonstrated to function as a tumor suppressor gene in renal cell carcinoma [10, 21]. Moreover, analysis of the inactivation rate in large-scale studies using clinical specimens revealed that SETD2 may occupy a third important place in ccRCC development, inferior only to VHL and PBRM1 [22]. Nevertheless, the mechanism underlying the inactivation of SETD2 in ccRCC still remains unclear. Generally, to resolve this type of problem, three aspects are concerned, including genetic mutation, epigenetic and post-transcriptional modulation. However, only 3% of ccRCC cases were detected with somatic truncated mutations of SETD2 gene [25], which was significantly less than VHL and PBRM1. In addition, aberrant hypermethylation of the SETD2 promoter was absent or rare in ccRCC [30]. Thus, the post-transcriptional regulation probably represents an important and unexplored area for the inactivation of SETD2 in ccRCC.

Currently, a number of microRNAs have been identified that regulate most human gene expression at the post-transcriptional level and have an important role in tumorigenesis [31–33]. It is well known that microRNAs are a group of small non-protein-coding RNAs (21–25 nucleotides) that regulate target genes in plants and animals [34]. MicroRNAs often bind to the 3′-untranslated region (UTR) of target mRNAs and constitute the RISC (RNA-induced silencing complex), which functions as a negative regulator via transcriptional or post-transcriptional silencing of target gene(s) [35]. Interestingly, increasing evidence has demonstrated that microRNAs are involved in the gene regulatory network [36]. On the one hand, a single miRNA can affect numerous genes, and on the other hand, several miRNAs may associate with the same target gene [37]. Moreover, they were verified to be involved in various cell signaling pathways and to affect proliferation, apoptosis, and differentiation, among other processes [38–40]. Numerous miRNAs have also been found in renal cell carcinoma in which some were identified to function as an oncogene, while others were demonstrated to play a tumor suppressor role [41–43]. Thus, in this study, we explored the post-transcriptional regulation of SETD2 by microRNAs in ccRCC.

## RESULTS

### Low levels of SETD2 were inversely correlated with endogenous miR-23b-5p, miR-34b-3p and miR-106b-5p in ccRCC tissues and cell lines

**Figure 1:**
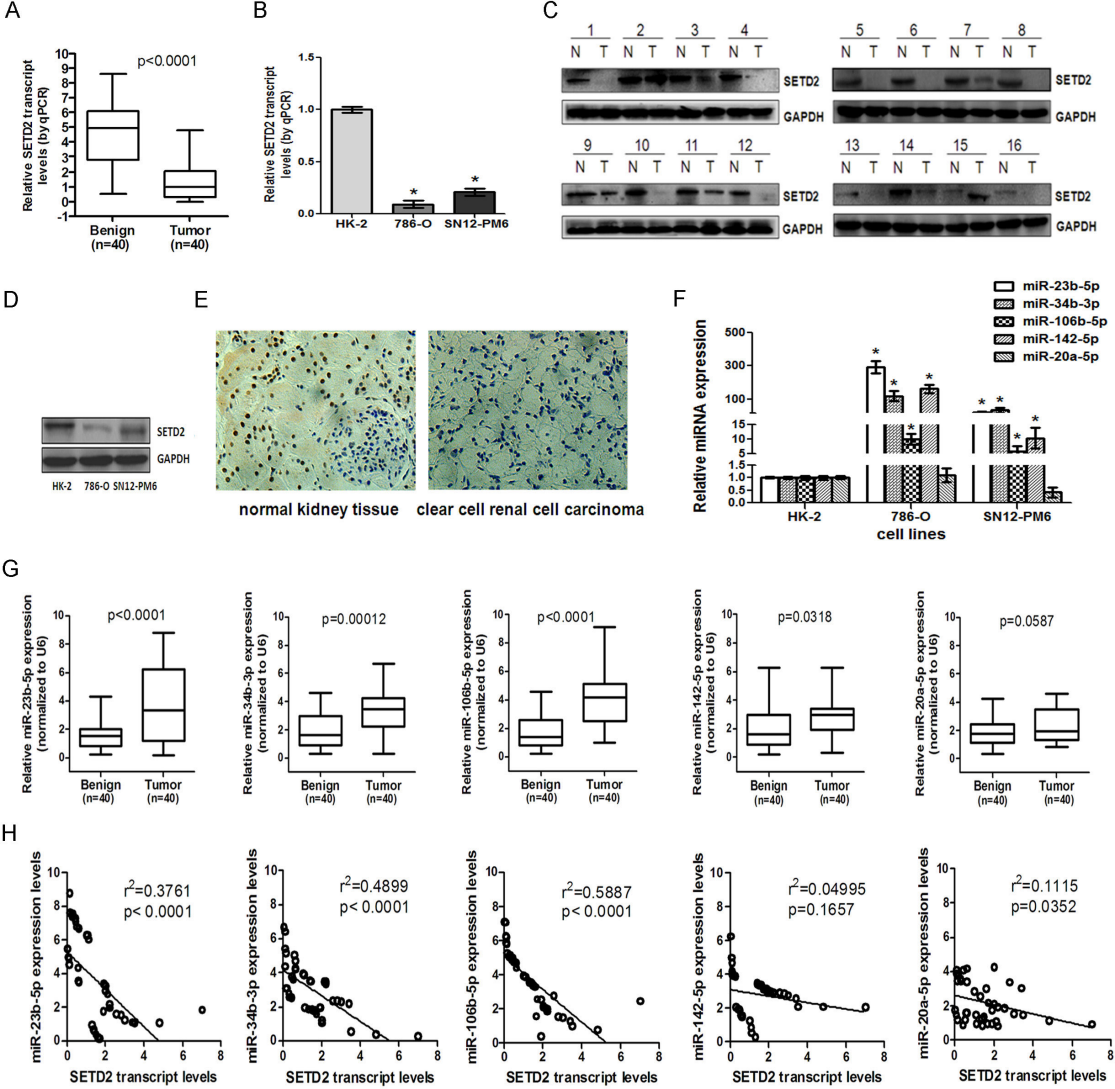
SETD2 was lowly expressed and inversely correlated with endogenous miR-23b-5p, miR-34b-3p and miR-106b-5p in ccRCC tissues and cell lines The expression of SETD2 mRNA in 40 pairs of human ccRCC samples **(A)** and human proximal tubule epithelial cell line HK-2 and ccRCC cell lines 786-O, SN12-PM6 **(B)** were examined by real-time RT-PCR, using GAPDH as the internal control. Western blot was used to detect the expression of SETD2 protein in human ccRCC samples **(C)** and HK-2, 786-O, and SN12-PM6 cells **(D)**. Immunohistochemistry was executed to check the SETD2 protein expression in ccRCC samples and surrounding normal kidney tissues (40 ×) **(E)**. The microRNAs including miR-23b-5p, miR-34b-3p, miR-106b-5p, miR-142–5p and miR-20a-5p were tested among HK-2,786-O and SN12-PM6 cell lines **(F)** as well as ccRCC tissues **(G)** by real-time RT-PCR, using U6 as an internal control. The linear regression between the expression of SETD2 mRNA and miRNAs in 40 ccRCC cases were analyzed respectively **(H)**. *P < 0.05 compared with HK-2 cells. Results are the means ± SD.

To determine the expression level of SETD2, we collected 40 pairs of primary ccRCC samples and surrounding normal kidney tissues. As shown in Figure 1A and 1C, both SEDT2 mRNA and protein levels were markedly reduced in ccRCC tissues as compared with that in matched normal tissues (*P* < 0.0001). Immunohistochemistry revealed that SETD2 was localized in the cell nucleus of normal renal tubular epithelial cells, and was strikingly decreased in ccRCC tissues (Figure 1E). Low levels of SETD2 were also detected in ccRCC 786-O and SN12-PM6 cell lines as compared with HK-2 cells (Figure 1B and 1D). In contrast, among the predicted miRNAs that might target SETD2, the expression of miR-23b-5p, miR-34b-3p, miR-106b-5p and miR-142–5p were significantly higher in ccRCC cell lines and tissues, while miR-20a-5p showed no significant difference (Figure 1G). Moreover, correlation analysis indicated that miR-23b-5p, miR-34b-3p and miR-106b-5p were inversely correlated with the expression of SETD2 in ccRCC (*p* < 0. 0001, Figure 1H).

### miR-106b-5p down-regulated SETD2 expression in ccRCC cells

**Figure 2:**
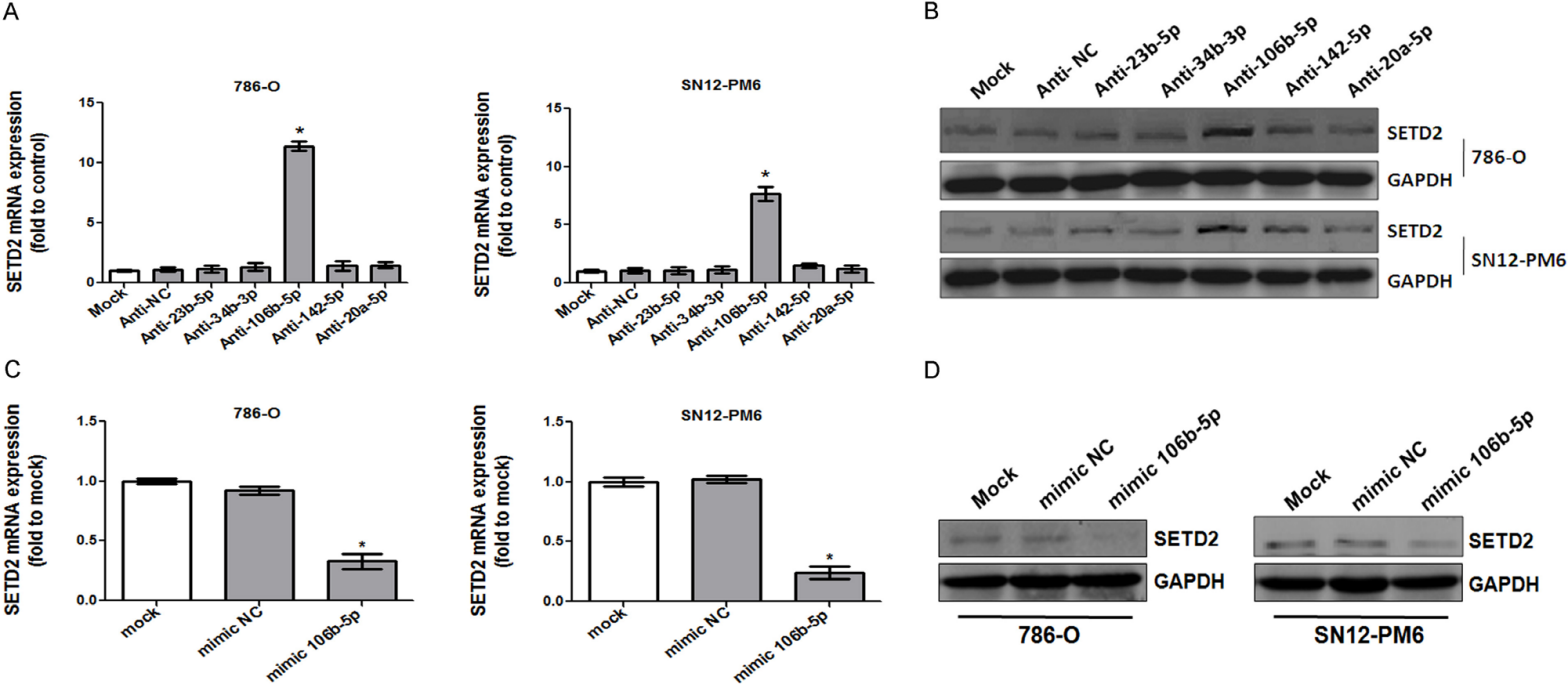
miR-106b-5p downregulated the expression of SETD2 in ccRCC cells 786-O and SN12-PM6 cells were transfected for 72 h with 100 nM anti-miR negative control, anti-miR-23b-5p, anti-miR-34b-3p, anti-miR-106b-5p, anti-miR-142–5p or anti-miR-20a-5p. SETD2 mRNA and protein expression levels were tested by real-time RT–PCR **(A)** and western blot **(B)**, respectively. 786-O and SN12-PM6 cells were transfected for 72 h with 50 nM negative control mimic or miR-106b-5 mimic, real-time RT–PCR and western blot were used to analyze the expression of SETD2 mRNA **(C)** and SETD2 protein **(D)**. *P < 0.05 versus anti-miR negative control, **P < 0.05 versus negative control mimic. Results are the means ± SD in triplicate.

To investigate whether the predicted microRNAs could regulate the expression of SETD2, we respectively transfected 100 nM synthesized antagomirs against miR-23b-5p, miR-34b-3p, miR-106b-5p, miR-142–5p and miR-20a-5p into 786-O as well as SN12-PM6 cells. As shown in Figure 2A, transfection of antagomir against miR-106b-5p significantly up-regulated SETD2 mRNA by 10.4- to 13.9-fold in 786-O cells, and 6.7- to 8.5-fold in SN12-PM6 cells, when compared with mock-transfected or negative control. The protein level of SETD2 was also significantly increased upon tranfection of antagomir against miR-106b-5p (4.4- to 7.5-fold in 786-O cells, 3.4- to 5.9-fold in SN12-PM6 cells) (Figure 2B). Meanwhile, neither SETD2 mRNA nor protein was affected upon transfection of the other antagomirs (Figure 2A and 2B). Furthermore, transfection of miR-106b-5p mimic resulted in the decreased mRNA and protein levels of SETD2 in 786-O and SN12-PM6 cells (Figure 2C and 2D). These results indicated that miR-106b-5p could down-regulate SETD2 expression in ccRCC cells.

### miR-106b-5p directly targeted the SETD2 mRNA 3′UTR

**Figure 3:**
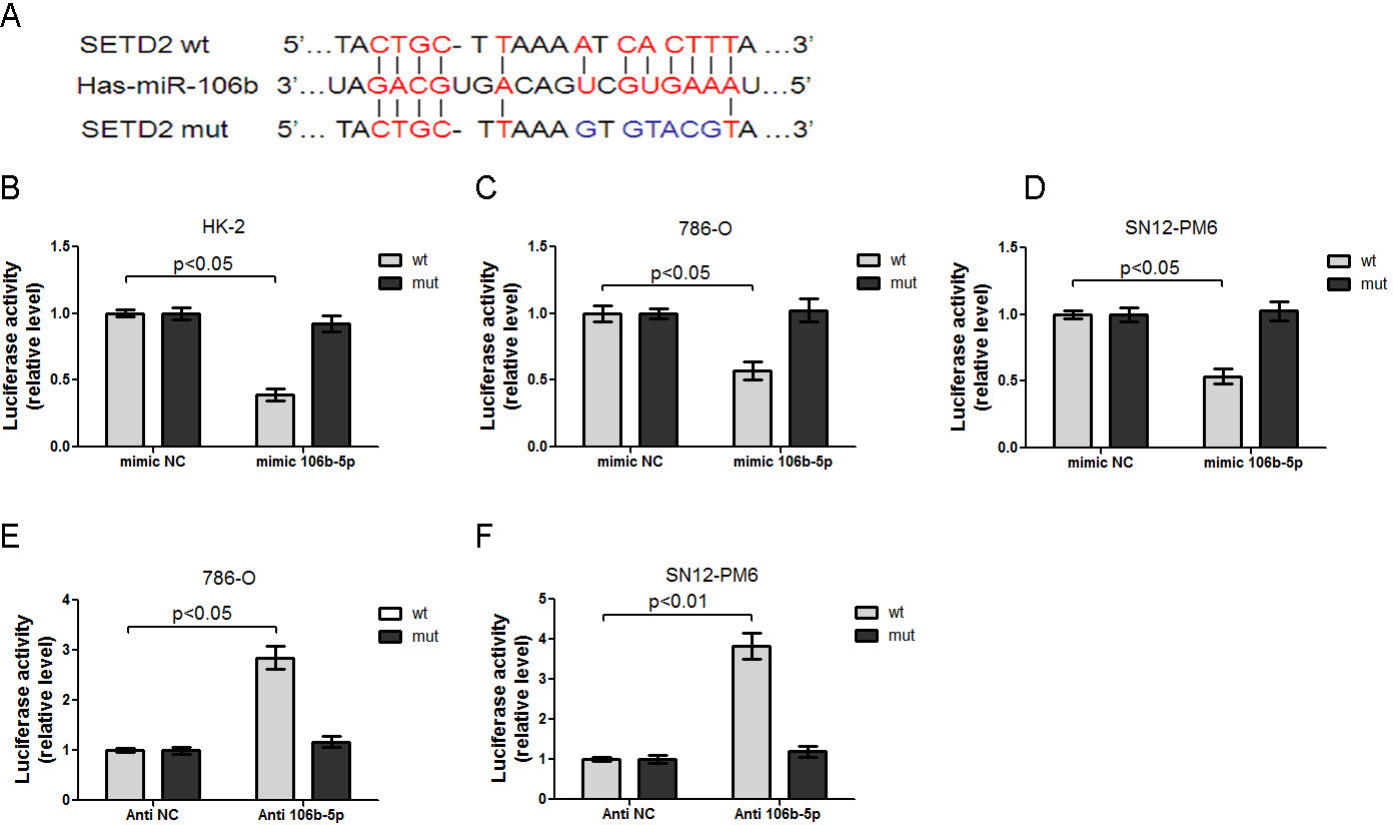
miR-106b-5p directly interacts with 3′UTR of SETD2 mRNA **(A)** Chematic representation of miR-106b-5p target binding site in the SETD2 mRNA 3′UTR identified by the microinspector prediction program. Wild type (wt) and mutation (mut) of 3′UTR in seed sequences were indicated. Transfection of miR-106b-5p mimic into HK-2 **(B)**, 786-O **(C)** and SN12-PM6 cells **(D)** resulted in decreased luciferase activities of SETD2 3′-UTR reporter, when compared with those transfected with mimic negative control (mimic NC). These effects were abolished by the mutation in the putative miR-106b-5p binding site within the 3′-UTR of SETD2. Transfection of anti-miR-106b-5p inhibitor (100 nM) into 786-O **(E)** and SN12-PM6 **(F)** cells increased the luciferase activity when compared with those transfected with negative control inhibitor (anti-NC), whereas mutation of miR-106b-5p recognition site abolished these effects. Results are the means ± SD in triplicate.

To confirm whether miR-106b-5p could repress the expression of SETD2 through directly interacting with its binding sites in the mRNA 3′-UTR, we amplified and cloned the full-length 3′UTR of the SETD2 or the mutation of miR-106b-5p seed recognition sequence into the pmiR-RB-REPORT™ Luciferase reporter vector (Figure 3A). The plasmids were co-transfected with miR-106B-5p mimic or negative control mimic, antagomir against miR-106b-5p or negative control antagomir, respectively. The renilla luciferase activities normalized to that of firefly were significantly reduced in HK-2, 786-O and SN12-PM6 cells transfected with miR-106B-5p mimic, and the effects were abolished by mutating the predicted miR-106b-5p binding site within the SETD2 mRNA 3′-UTR, indicating a specific suppressive effect of miR-106b-5p on SETD2 (Figure 3B, 3C, and 3D). Moreover, knockdown of miR-106b-5p with miR-106b-5p antagomir increased the luciferase activity in 786-O and SN12-PM6 cells, whereas mutation of miR-106b-5p recognition site abolished these effects (Figure 3E and 3F). These results collectively demonstrate that SETD2 is indeed a direct target of miR-106b-5p.

### Attenuation of miR-106b-5p suppressed ccRCC cells proliferation through up-regulation of SETD2

**Figure 4:**
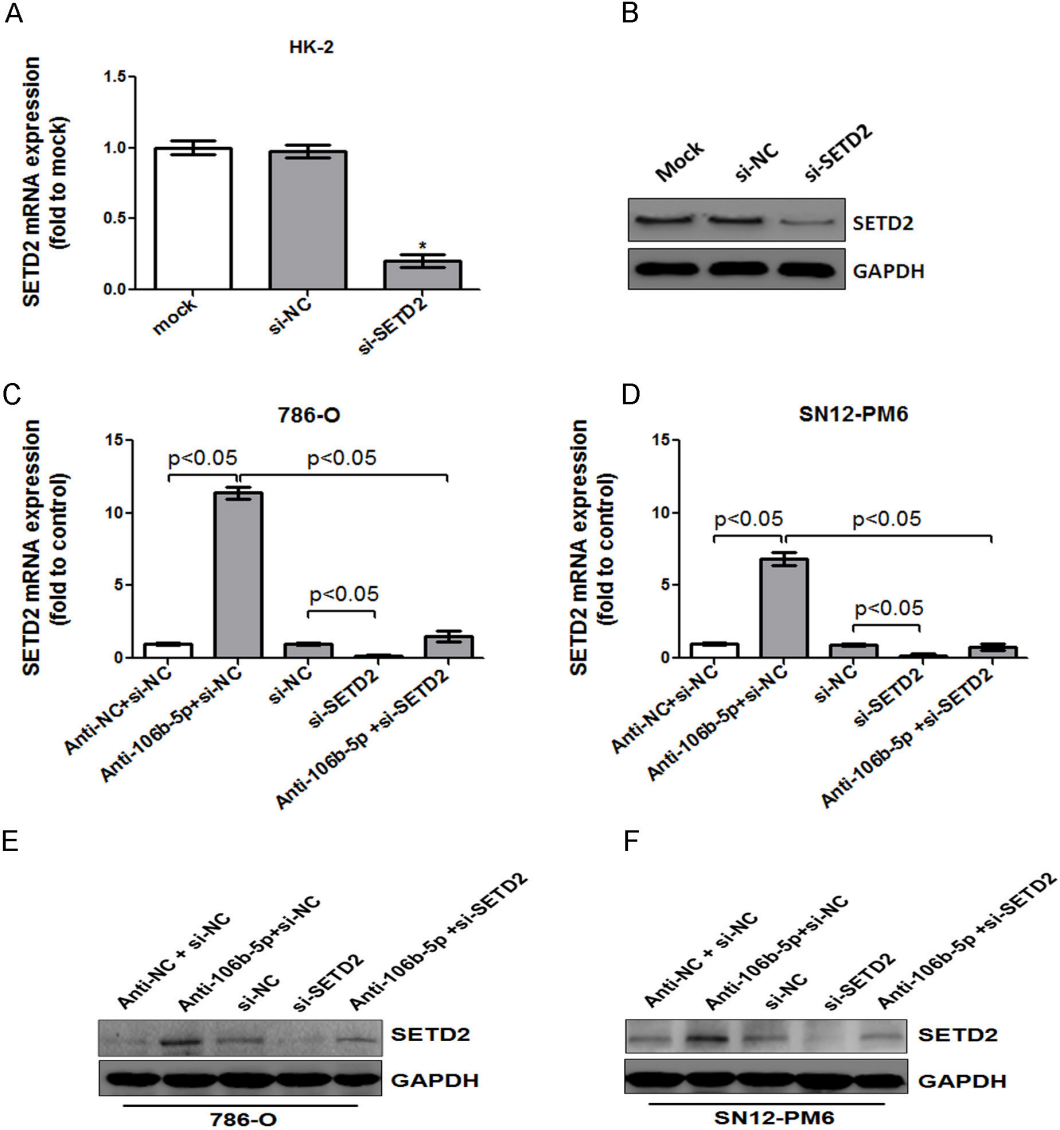
MiR-106b-5p antagomir induced up-regulation of SETD2 expression was reversed by knockdown of SETD2 in ccRCC cells The effects of si-SETD2 on SETD2 expression were confirmed first in HK-2 cells through real-time PCR **(A)** and western blot **(B)** respectively. Transfection of antagomir against miR-106b-5p resulted in the increased level of SETD2 mRNA in both 786-O **(C)** and SN12-PM6 **(D)** cells, which was blocked by co-transfection of si-SETD2. The changes of SETD2 protein levels in both 786-O **(E)** and SN12-PM6 **(F)** cells were further confirmed by Western blot. *P < 0.05 versus negative control. Results are the means ± SD in triplicate.

**Figure 5:**
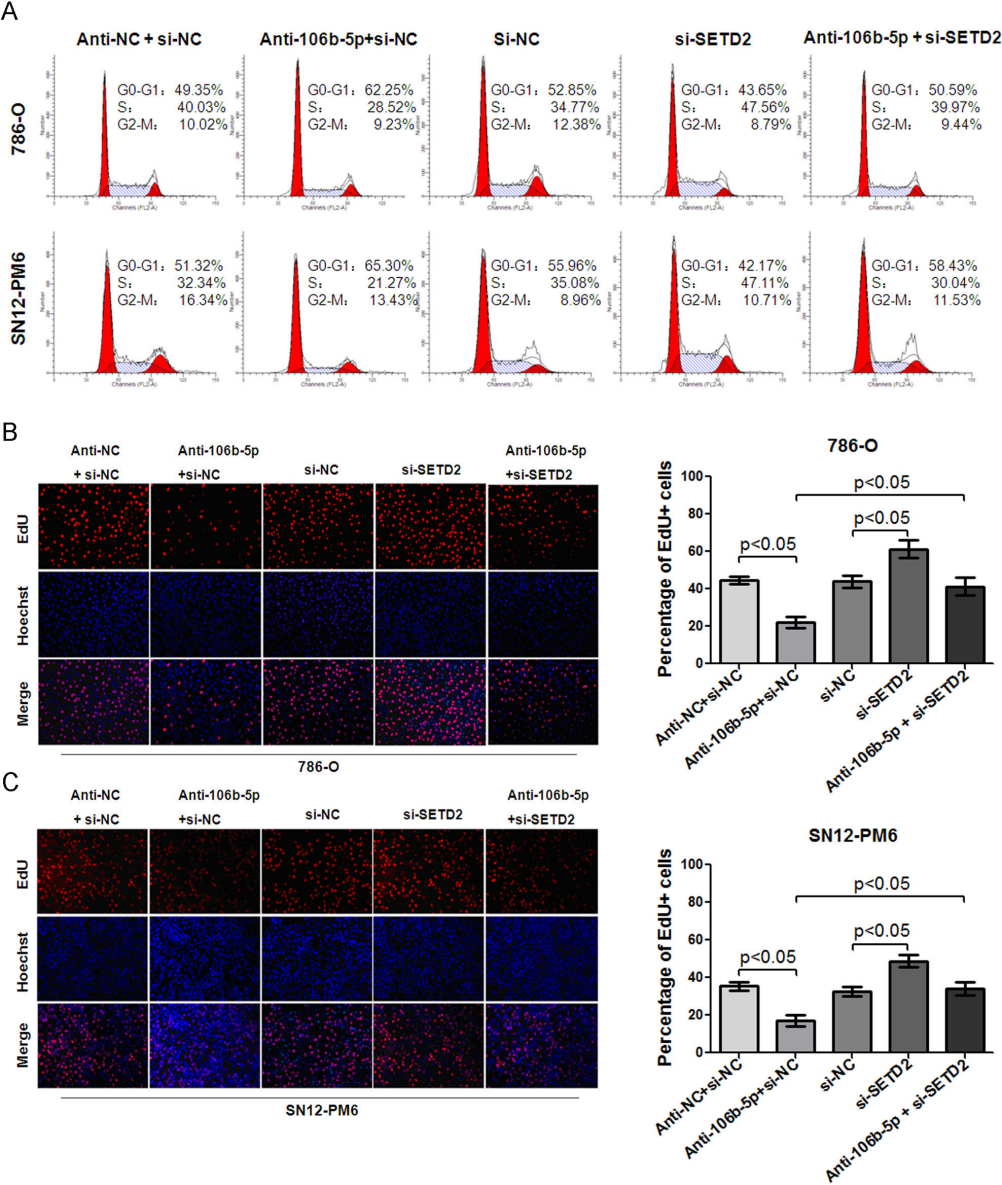
MiR-106b-5p antagomir induced cell cycle arrest and proliferation suppression through up-regulation of SETD2 expression in ccRCC cells **(A)** Flow cytometry indicated that transfection of miR-106b-5p antagomir resulted in cell cycle arrest at G0/G1 phase in both 786-O and SN12-PM6 cells compared with negative control ( *p* < 0.05), while transfection of si-SETD2 decreased the basal rate of cells in G0/G1 phase and effectively reversed the miR-106b-5p antagomir induced G0/G1 phase arrest (*p* < 0.05). EdU assay showed that transfection of miR-106b-5p antagomir inhibited proliferation of 786-O **(B)** and SN12-PM6 **(C)** cells compared with negative control (* p* < 0.05), which was reversed by the transfection of si-SETD2. Results are the means ± SD in triplicate.

Given that SETD2 plays a tumor suppressor role and miR-106b-5p has been found responsible for post-transcriptional inhibition of SETD2 in our study, we further explored whether attenuation of miR-106b-5p could suppressed ccRCC cell biologic activity through up-regulation of SETD2. siRNA targeting the coding region of SETD2 (si-SETD2) was designed. As shown in Figure 4A and 4B, both the mRNA and protein levels of SETD2 were significantly silenced in HK-2 cells after transfection of si-SETD2. The knockdown effects were further confirmed in 786-O and SN12-PM6 cells, and si-SETD2 could reverse the miR-106b-5p antagomir induced up-regulation of SETD2 mRNA and protein (Figure 4C–4F). The cell cycle assay indicated that attenuation of miR-106b-5p induced cell cycle arrest at G0/G1 phase in 786-O and SN12-PM6 cells compared with negative control (Figure 5A). Meanwhile, transfection of si-SETD2 both decreased the basal rate of cells in G0/G1 phase and effectively reverse the miR-106b-5p antagomir induced G0/G1 phase arrest (Figure 5A). Moreover, the EdU assay showed that attenuation of miR-106b-5p inhibited proliferation of 786-O and SN12-PM6 cells, which was also reversed by transfection of si-SETD2 (Figure 5B and 5C). These observations revealed that attenuation of miR-106b-5p was able to suppress cell proliferation through up-regulation of SETD2 in ccRCC cells.

### Attenuation of miR-106b-5p promoted processing of caspase-3 and induced ccRCC cells apoptosis through up-regulation of SETD2

**Figure 6:**
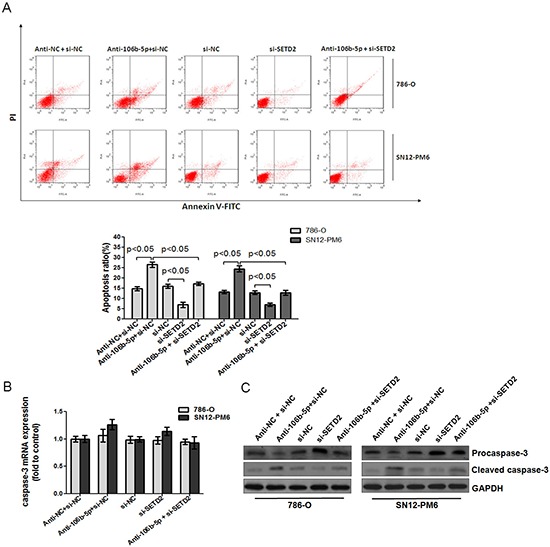
Attenuation of miR-106b-5p promoted caspase-3 mediated apoptosis through up-regulation of SETD2 expression **(A)** Annexin V and PI staining flow cytometry assay revealed that transfection of miR-106b-5p antagomir increased the rate of apoptosis in 786-O and SN12-PM6 cells compared with negative control group, while transfection of si-SETD2 decreased the apoptotic cells, and significantly inhibited miR-106b-5p antagomir induced cell apoptosis. **(B)** The effects of miR-106b-5p antagomir, si-SETD2 and their cotransfection on the expression of caspase-3 mRNA in 786-O and SN12-PM6 cells were examined by real-time RT-PCR. **(C)** The effects of miR-106b-5p antagomir, si-SETD2 and their cotransfection on the processing of caspase-3 were detected by Western blot. Results are the means ± SD in triplicate.

To further study the inhibitory effect of miR-106b-5p antagomir on ccRCC cells, we examined cell apoptosis by flow cytometry using Annexin V and PI staining method. The number of total apoptotic cells was significantly increased upon transfection of miR-106b-5p antagomir in 786-O and SN12-PM6 cells compared with negative control group (Figure 6A). In contrast, transfection of si-SETD2 decreased the apoptotic cells, and significantly inhibited miR-106b-5p antagomir induced cell apoptosis (Figure 6A). Caspase-3 is the essential executioner of apoptosis. Transfection of miR-106b-5p antagomir produced processing of caspase-3 precursors to their active products, while transfection of si-SETD2 not only decreased the basal level but also reversed the miR-106b-5p antagomir induced high level of active caspase-3 (Figure 6C). Meanwhile, the mRNA level of caspase-3 was not affected (Figure 6B). These data indicated that attenuation of miR-106b-5p could promote processing of caspase-3 and induce ccRCC cells apoptosis through up-regulation of SETD2.

### Inhibition of miR-106b-5p enhanced the binding of H3K36me3 to the promoter of p53, and upregulated p53 transcription through a SETD2 dependent way

**Figure 7:**
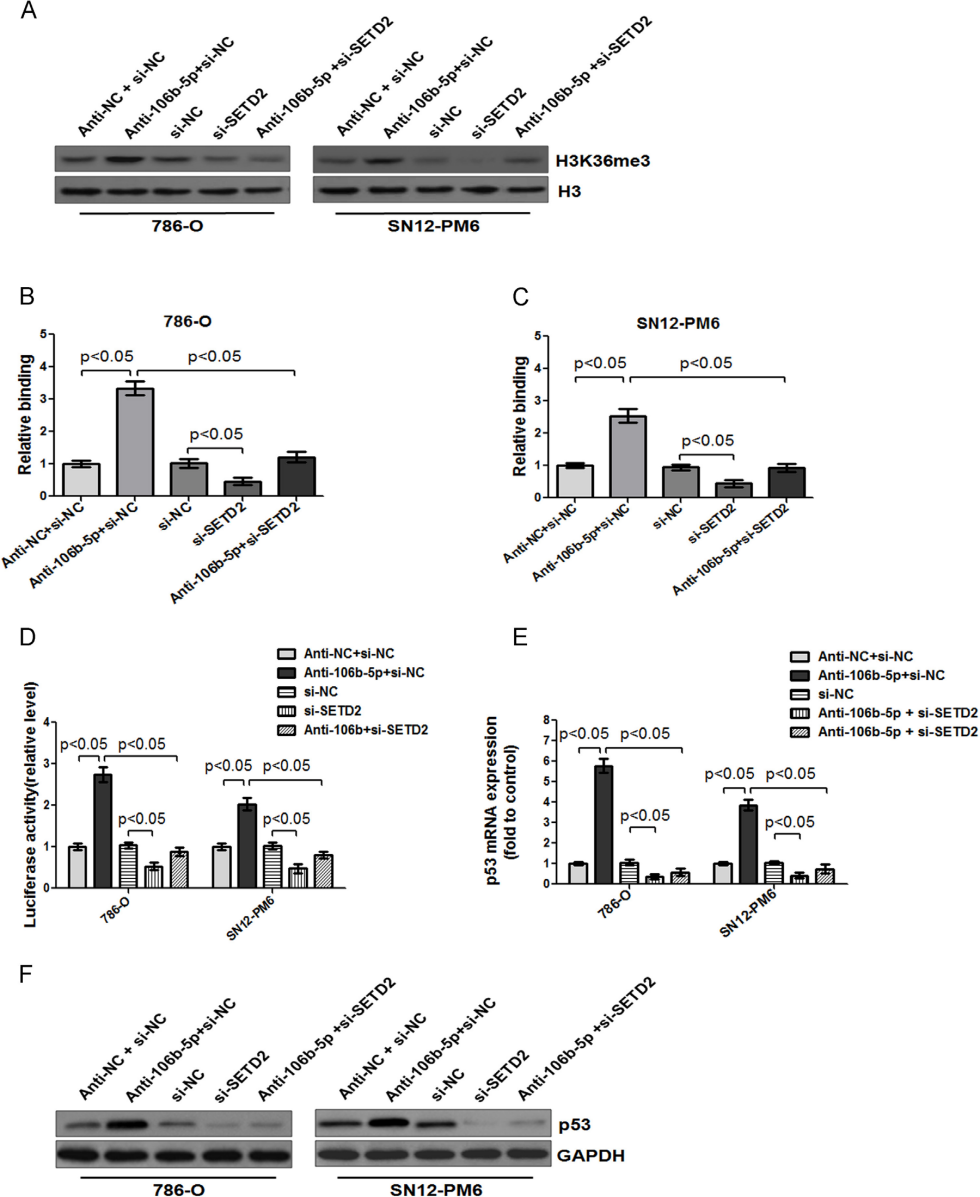
Inhibition of miR-106b-5p enhanced the binding of H3K36me3 to the promoter of p53, and upregulated p53 transcription through a SETD2 dependent way **(A)** Western blot showed that transfection of miR-106b-5p antagomir up-regulated the level of H3K36me3 in 786-O and SN12-PM6 cells compared with negative control group, which was reversed by cotransfection of si-SETD2. ChIP assay indicated that transfection of miR-106b-5p antagomir enhanced binding of H3K36me3 to the promoter of p53 in 786-O **(B)** and SN12-PM6 **(C)** cells, which was abolished by cotransfection of si-SETD2. **(D)** Luciferase activity assay demonstrated that a significant increase of p53 promoter activity was induced by the attenuation of miR-106b-5p, while it was reversed by cotransfection of si-SETD2 in 786-O and SN12-PM6 cells. Real-time RT-PCR and western blot analysis indicated that transfection of miR-106b-5p antagomir increased the p53 expression in both mRNA **(E)** and protein **(F)** levels, whereas knockdown of SETD2 reversed the effects. Results are the means ± SD in triplicate.

Different from DNA methyltransferase (DNMT) such as DNMT1, DNMT 3A/B that facilitate the DNA methylation, SETD2 has been identified as a histone methyltransferase that could convert histone H3 lysine 36 (H3K36) into trimethylated H3K36 (H3K36me3) of nucleosomes positioned on active genes [25, 44, 45]. Tumor suppressor gene p53, which is critically involved in cell cycle regulation and apoptosis, has been found rarely mutated in ccRCC [46]. Therefore, we further investigated whether miR-106b-5p antagomir induced up-regulation of SETD2 could enhance the binding of H3K36me3 to the promoter of p53. As shown in Figure 7A, transfection of miR-106b-5p antagomir significantly increased the total H3K36me3 levels in 786-O and SN12-PM6 cells, which was inhibited by cotransfection with si-SETD2. Moreover, ChIP assay showed that the binding of H3K36me3 to the promoter of p53 was also enhanced upon transfection of miR-106b-5p antagomir, while transfection of si-SETD2 not only decreased the basal level but also reversed the miR-106b-5p antagomir induced binding of H3K36me3 (Figure 7B and 7C). Consistent with these findings, transfection of miR-106b-5p antagomir resulted in the increased promoter activity (Figure 7D), mRNA (Figure 7E) and protein (Figure 7F) levels of p53, and the effects were abolished by cotransfection with si-SETD2. These results demonstrated that inhibition of miR-106b-5p could effectively enhance the binding of H3K36me3 to the promoter of p53, resulting in the up-regulation of its transcription through a SETD2 dependent way. Thus, p53 probably plays an important role in attenuation of miR-106b-5p to suppress cell biologic activity of ccRCC.

## DISCUSSION

miR-106b-5p is transcribed from the miR-106b-25 cluster, which is located on human chromosome 7q21, and there have been many pioneering studies based on its tumor-specificity [47, 48]. Its over-expression in hepatocellular carcinoma promoted cell migration and metastasis by activating epithelial-mesenchymal transition [49]. A microarray study demonstrated that miR-106b-5p was up-regulated in colon cancer with lymph node metastasis [50]. Another study reported that miR-106b-5p played an oncogenic role in esophageal neoplasms, and p21 was identified as the target gene of miR-106b-5p [51]. In gastric cancer, miR-106b-5p could promote the cell cycle by down-regulating target gene expression of p21 and E2F5 [52]. Over-expression of miR-106b-5p was also demonstrated in breast cancer, and the miR-106b/MMP2/ERK pathway might play a pivotal role in bone metastasis of breast cancer [53]. In ccRCC, over-expression of miR-106b-5p was confirmed in renal cancer tissue and has been demonstrated to function as a potential marker for early metastasis after nephrectomy [54]. However, the function of miR-106b-5p in ccRCC cells is still unknown. In the present study, our data showed that miR-106b-5p was aberrantly up-regulated in both ccRCC samples and cell lines. Moreover, we found that attenuation of miR-106b-5p suppressed cell proliferation and induced cell apoptosis in 786-O and SN12-PM6 ccRCC cells, suggesting it was critical in regulating ccRCC cell biologic activity. Thus, miR-106b-5p is probably a potential candidate for the therapeutics of ccRCC.

SETD2, a methyltransferase to trimethylate H3K36 of nucleosomes positioned on active genes, has recently been verified to function as a tumor suppressor gene in ccRCC. However, the mechanism underlying the inactivation of SETD2 in ccRCC, especially the post-transcriptional regulation, still remains unclear. Here, the results revealed that miR-106b-5p was inversely correlated with the expression of SETD2 in ccRCC tissues. Moreover, miR-106b-5p could directly target the SETD2 mRNA 3′UTR and down-regulate both mRNA and protein of SETD2, suggesting that miR-106b-5p may regulate SETD2 expression by inducing mRNA degradation and/or translational suppression. Therefore, our data demonstrate that SETD2 is a novel target of miR-106b-5p, which has not been reported previously. More interestingly, the present findings reveal that the post-transcriptional regulation of SETD2 by miR-106b-5p accounts for its inactivation in ccRCC. Previous reports have shown the potential role of SETD2 inactivation in carcinogenesis and progression [10, 29], and SETD2 is required for regulating the p53-dependent cell-cycle checkpoint and cell survival following DNA damage in ccRCC cells [55]. In this study, the results showed that knockdown of SETD2 decreased the rate of ccRCC cells in G0/G1 phase, and reduced the apoptotic cells. Furthermore, the inhibition of ccRCC cell proliferation and the induction of apoptotic cells by attenuation of miR-106b-5p were reversed upon knockdown of SETD2. Thus, our experimental evidences reveal that SETD2 plays an important role in regulating ccRCC cell proliferation and apoptosis, and loss of SETD2 affords for the miR-106b-5p-mediated oncogenic role.

The regulatory mechanism downstream of SETD2 still remains unclear. Previous studies demonstrated that SETD2 was responsible for the trimethylation of H3K36 in a non-redundant manner, which was highly associated with the activation of euchromatin transcription and specific genes, such as tumor suppressor genes, which might exhibit transcriptional activation [25, 26]. In our study, the H3K36me3 level was indeed increased in ccRCC cells upon transfection of miR-106b-5p antagomir, which was reversed by cotransfection with si-SETD2. To further explore the relationship among SETD2, H3K36me3 and p53, we performed ChIP and luciferase activity analyses of the p53 promoter to obtain a comprehensive evaluation. The results indicated that the binding of H3K36me3 to the promoter of p53 were enhanced upon transfection of miR-106b-5p antagomir, resulting in the increased promoter activity, mRNA and protein levels of p53. These effects were abolished by cotransfection with si-SETD2. It has been reported that SETD2 could interact with p53 and selectively regulate the p53 downstream genes, including puma, noxa, p53AIP1, fas, p21, tsp1, and huntingtin [56]. SETD2 was also required for DNA double-strand break repair and p53 phosphorylation and activation in ccRCC cells [55]. Our data further revealed that SETD2 could increase the binding of H3K36me3 to the promoter of p53 and enhance p53 activity at mRNA transcription level, providing a novel SETD2-depedent role in regulating the function of p53. These results indicated p53 probably plays a critical role in attenuation of miR-106b-5p to suppress cell biologic activity through SETD2.

In conclusion, we have demonstrated that miR-106b-5p is inversely correlated with SETD2 in ccRCC tissues. Moreover, miR-106b-5p suppresses the expression of SETD2 via the binding site in the 3′-UTR in ccRCC cells, and plays an important role in regulating ccRCC cell proliferation and apoptosis through SETD2-dependent way. In addition, SETD2 increases the binding of H3K36me3 to the promoter of p53 and enhances its transcription. This study extends the knowledge about the regulation of SETD2 at the posttranscriptional level by miRNA and regulatory mechanism downstream of SETD2, suggesting that miR-106b-5p may be of potential values as novel candidate for the therapeutics of ccRCC.

## MATERIALS AND METHODS

### Cell lines and human tissue specimens

Human ccRCC cell line 786-O was obtained from American Type Culture Collection (ATCC, Manassas, VA, USA) and maintained in RPMI 1640 medium (Gibco, Grand Island, NY, USA). The human ccRCC cell line SN12-PM6 was a gift from Dr. X.P. Zhang (Department of Urology, Union Hospital, Wuhan, China) and was cultured in DMEM medium (Gibco). The human proximal tubule epithelial cell line HK-2 was also obtained from ATCC and maintained in complete medium consisting of keratinocyte serum-free medium (K-SFM) supplemented with bovine pituitary extract (BPE), and human recombinant epidermal growth factor (EGF) (Invitrogen, Carlsbad, CA, USA). Forty pairs of primary clear cell renal cell carcinoma samples and surrounding normal kidney tissues were obtained from patients who underwent partial or radical nephrectomy at Department of Urology of the Union Hospital of Tongji Medical College between 2012 and 2013. All specimens were immediately snap-frozen in liquid nitrogen after surgical removal. Histological and pathological diagnoses were confirmed by a pathologist. All specimens were obtained with appropriate informed consent from the patients and a grant obtained from the Medical Ethics Committee of China.

### Target prediction and bioinformatics analysis

The online algorithms Target-Scan, microRNA.org and miRWalk were used to predict miRNAs that might target SETD2. The miRNA microarray analysis was available according to a previous study of clear cell renal cell carcinoma by Hans-Joachim and Lawrie et al., and the data are available at the Gene Expression Omnibus (GEO) repository database [http://www.ncbi.nlm.nih.gov/geo/, accession number GSE12105 and GSE51554]. Finally, miR-23b-5p, miR-34b-3p, miR-106b-5p, miR-142–5p, and miR-20a-5p were selected to be investigated in our study.

### Real-time RT–PCR analysis of miRNAs and mRNA expression

Total RNA was extracted from tissue and cell lines using TRIzol reagent (Invitrogen). For SETD2 mRNA analysis, complementary DNA was synthesized using the reverse transcription kit PrimeScript™ RT reagent Kit with gDNA Eraser (Takara Biomedical Technology, Dalian, China). For miRNA detection, RNA was reverse transcribed into cDNA using the SYBR® PrimeScript™ miRNA RT-PCR Kit (Takara Biomedical Technology). The SYBR® Premix Ex Taq™ (Tli RNaseH Plus) kit (Takara Biomedical Technology) was then used for real-time PCR applications. The primer set for SETD2 was 5′-AAC GGGAGGCTCAGAAACAA-3′(forward) and 5′-GTGGGTAACCAGCAAAGGG A-3′ (reverse). The primer set for p53 was 5′-GCTTTGAGGTGCGTGTTTGT-3′ (forward) and 5′-GTTTCTTCTT TGGCTGGGGA-3′ (reverse). GAPDH served as an internal control using primers 5′-TCAAGAAGGTGGTGAAGCAG-3′(forward) and 5′-CGTCAAAGGTGGAG GAGTG-3′(reverse). The primers of five miRNAs were purchased from Guangzhou RiboBio (RiboBio, Guangzhou, China), and U6 was used as an internal control for the detection of miRNAs. All analyses were performed using the StepOnePlus Real-Time PCR System (Applied Biosystems, Foster City, CA, USA). The ΔΔCT method was used to calculate the relative expression of different genes.

### RNA oligos synthesis and transfection

The miRNA inhibitors (anti-miR-23b-5p, anti-miR-34b-3p, anti-miR-106b-5p, anti-miR-142–5p, anti-miR-20a-5p), miRNA inhibitor negative control, miR-106b-5p mimic and negative control mimic were designed and synthesized by RiboBio. 786-O and SN12-PM6 cells were transiently transfected with the above miRNA inhibitors and mimics. The siRNA targeting SETD2 mRNA and siRNA negative control were also obtained from RiboBio. The sequence of siSETD2 is: sense: 5′-GCAGGACA CUAUAUCUAAU dTdT-3′; anti-sense: 3′-dTdT CGUCCUGUGAUAUAGAUUA-5′. Twenty-four hours prior to transfection, cells were plated in a 6-well plate (Corning, New York, NY, USA) at 40–60% confluence. Transfection was then performed using Lipofectamine 2000 (Invitrogen) according to the manufacturer's protocol. The medium was replaced 4–6 h after transfection with new culture medium. The transfection efficiency was evaluated using Cy3-labeled oligonucleotides as a negative control (data not shown).

### Plasmid construction and luciferase reporter assay

The 3′-UTR of the SETD2 gene was amplified from genomic DNA of HK-2 cells using PCR, which was then gel purified, digested and cloned into the pmiR-RB-REPORT™ Luciferase vector reporter (RuiBio) between the XhoI and NotI sites. The reporter vector contains a hluc (synthetic firefly luciferase gene), which encodes firefly luciferase as an internal control, and hRluc (synthetic Renilla luciferase gene), which encodes Renilla luciferase as the reporter. The amplified PCR product is based on the primers: 5′-GCCCTCGAGCTGTTGGGCCAGGGTGGGA G-3′ (forward) and 5′-GTTTGCGGCCGCTTAACTTTTAGGGAACACACATGCC-3′ (reverse). A mutation of the miR-106b-5p binding site in the 3 ‘UTR of SETD2 mRNA was generated using site-directed mutagenesis by the megaprimer PCR method with primers 5′-AATATGCGGCCGCCACATGCTACTGCTTAAAAT-3′ (forward) and 5′-GCCGAGCTCGTGTACGTACTTTATCCAA-3′ (reverse). The mutated scrambled sequences were prepared using the online software (http://www.genscript.com/ssl-bin/app/scramble). The mutated PCR product was also inserted into the cloning site of the pmiR-RB-REPORT™ Luciferase vector reporter (RuiBio). All of the cloned products were cofirmed by final sequencing. All primers were designed and synthesized by Guangzhou RiboBio (RuiBio), and the primer sequences are available upon request. The p53 promoter (1121 bp) was cloned into the pGL-3 plasmid, which was a kind gift from M.F. Wu (Divisions of Medical Oncology and Chest Medicine, Chung Shan Medical University Hospital, Taichung City, Taiwan). The pGL-3 basic plasmid was conserved by our group.

### Western blotting analysis

Total protein from tissues and cell lines was extracted using RIPA (Thermo Scientific, Rockford, IL, USA), and nuclear protein was prepared using the Nucleoprotein Extraction Kit (Sangon Biotech, Shanghai, China) in the presence of protease inhibitor cocktail and PMSF (Beyotime Institute of Biotechnology, Haimen, China). The concentration of various protein samples was determined using the BCA Protein assay kit (Beyotime Institute of Biotechnology) according to the manufacturer's instructions. Thirty micrograms of each lysate was electrophoresed in 10% SDS-PAGE gels and then transferred onto PVDF membranes and detected using the ECL kit (Beyotime Institute of Biotechnology). The SETD2 (ab69836), p53 (ab28) and H3K36me3 (ab9050) antibodies were purchased from Abcam (Cambridge, MA, USA). GAPDH (D16H11), Caspase-3 (#9662) and cleaved Caspase-3 (#9664) antibodies were obtained from Cell Signaling Technology Inc. (Vebery, MA, USA). H3 antibody was ordered from Beyotime Institute of Biotechnology. Anti-rabbit and anti-mouse IgG secondary antibodies conjugated with horseradish peroxidase were provided by Wuhan Boster Bio-engineering Limited Company (Wuhan, China). ImageJ Software version 1.36b was applied to analyze the expression levels of different proteins.

### Immunohistochemistry

Immunostaining was performed on clear cell renal cancer tissue sections that had been previously confirmed for the pathological pattern by a pathologist. The avidin-biotin-peroxidase method was performed to examine the expression and location of the target gene. The primary antibody SETD2 was used at a dilution of 1:100. An Olympus microscope was used to obtain images.

### Flow cytometry analysis of the cell cycle and apoptosis

786-O and SN12-PM6 cells were transfected with the anti-miR negative control, anti-miR-106b-5p and SETD2 siRNA. Cells were harvested after 72 h and stained with propidium iodide (Sigma, St Louis, MO, USA) for cell cycle analysis. The ModFit LT software was used to analyze the data. For cell apoptosis, flow cytometry was also performed for detection using the Annexin V-PI apoptosis detection kit (BD Pharmingen, San Diego, CA, USA) according to the manufacturer's protocol.

### 5-ethynyl-20-deoxyuridine assay (EdU) Assay

786-O and SN12-PM6 cells were seeded in 96-well plates and transfected with oligoribonucleotides. After 48 h of transfection, EdU (Cell Light EdU DNA imaging Kit, RiboBio) was used to determine cell proliferation viability. EdU (100 μM) was added into the medium and cultured with the cells for 2 h. Next, images were obtained and analyzed using a microscope (Olympus, Tokyo, Japan). The positive cells were shown in red fluorescence compared with blue fluorescence of Hoechst-stained cells. The rate of EdU add-in cells/Hoechst-stained cells × 100% was used to calculate the cell proliferation activity.

### Chromatin immunoprecipitation (ChIP) Assay

The ChIP assay was performed according to the manufacturer's protocol using the EZ-ChIP kit (Upstate Biotechnology, Lake Placid, NY, USA). Real-time PCR primers for the p53 promoter were designed using the Premier Primer 5.0 software to amplify the adjacent regions surrounding the transcription start site, −92 to +100 (F: 5′-TTGTGCCAGGA GCCTCGC-3′, R: 5′-CAGGGAAGCGTG TCACCGT-3′). The SYBR® Premix Ex Taq™ kit was used for real-time PCR amplification using the StepOnePlus Real-Time PCR System. The amount of immunoprecipitated DNA was assessed by generating a standard curve and normalized against the anti-miR negative control group as well as the siRNA negative control.

### Statistical analysis

Data were analyzed using SPSS 17.0 software (SPSS Inc., Chicago, IL, USA). Paired t-tests were performed to compare the difference between paired tissues using real-time PCR and western blotting analyses. ANOVA analysis was followed among the different groups. The Chi-square test was performed to calculate the difference in immunohistochemistry. In addition, the Spearman correlation was used to determine the correlation between miRNA expression levels and SETD2 expression levels in tissue specimens. Statistically significant differences were established at *p* < 0.05.
